# Human-Induced Pluripotent Stem Cell Technology and Cardiomyocyte Generation: Progress and Clinical Applications

**DOI:** 10.3390/cells7060048

**Published:** 2018-05-25

**Authors:** Angela Di Baldassarre, Elisa Cimetta, Sveva Bollini, Giulia Gaggi, Barbara Ghinassi

**Affiliations:** 1 Department of Medicine and Aging Sciences, University “G.d’Annunzio” of Chieti-Pescara, 66100 Chieti, Italy; angela.dibaldassarre@unich.it (A.D.B.); giulia.gaggi@unich.it (G.G.); 2 Department of Industrial Engineering (DII), University of Padova, 35131 Padova, Italy; elisa.cimetta@unipd.it; 3 Laboratory of Regenerative Medicine, Department of Experimental Medicine, University of Genova, 16121 Genova, Italy; sveva.bollini@unige.it

**Keywords:** regenerative medicine, reprogramming, cardiac differentiation, secretoma, tissue engineering

## Abstract

Human-induced pluripotent stem cells (hiPSCs) are reprogrammed cells that have hallmarks similar to embryonic stem cells including the capacity of self-renewal and differentiation into cardiac myocytes. The improvements in reprogramming and differentiating methods achieved in the past 10 years widened the use of hiPSCs, especially in cardiac research. hiPSC-derived cardiac myocytes (CMs) recapitulate phenotypic differences caused by genetic variations, making them attractive human disease models and useful tools for drug discovery and toxicology testing. In addition, hiPSCs can be used as sources of cells for cardiac regeneration in animal models. Here, we review the advances in the genetic and epigenetic control of cardiomyogenesis that underlies the significant improvement of the induced reprogramming of somatic cells to CMs; the methods used to improve scalability of throughput assays for functional screening and drug testing in vitro; the phenotypic characteristics of hiPSCs-derived CMs and their ability to rescue injured CMs through paracrine effects; we also cover the novel approaches in tissue engineering for hiPSC-derived cardiac tissue generation, and finally, their immunological features and the potential use in biomedical applications.

## 1. Introduction

Cardiovascular disease (CVD) and heart failure (HF) still represent the major causes of mortality and morbidity in the Western world [1]. CVD and HF can arise from myocardial infarction (MI) [2], chemotherapy-derived cardiotoxicity [3], and congenital defects [4] affecting cardiac function. The pathological basis is mainly related to the very limited ability of the heart to withstand injury and aging, which is due to insufficient cardioprotection combined with almost the complete lack of myocardial renewal. In such scenarios, cardiac transplantation still represents the ultimate therapeutic option for HF, although it is severely hindered by the short supply of available donor hearts. This also translates into an economic burden for national health institutions, as more than a million hospitalizations due to HF are annually reported in the EU alone [4]. Cell-based cardiac tissue engineering strategies could provide regenerative therapeutic options and if these strategies utilize autologous cells, the limitations derived from biocompatibility and immune response would be surmounted. Recently, the development of reprogramming technology in 2006 in Yamanka’s lab [5] and the knowledge acquired in the cardiac specification and differentiation makes the potential replacement of the lost cardiomyocytes (CMs) more feasible. Indeed, the ability of human-induced pluripotent stem cells (hiPSCs) to differentiate into autologous tissue-specific cells, similar to embryonic stem cells (ESC), but without the need to destroy a human embryo, is an important breakthrough in human stem cell biology [6]. A number of pre-clinical studies have explored the effects of intramyocardial injection of hiPSCs derived cardiomyocytes into murine and porcine models of MI (a complete recent list of pre-clinical studies is provided in Lalit et al. [7]). Nelson et al. [8] showed that the intramyocardial injection of iPSC-derived CMs into a murine model of acute MI determined an improvement in the clinical outcomes four weeks after permanent coronary artery ligation. Thereby, hiPSCs have demonstrated significant potential as a tool in regenerative medicine.

Here, we review the recent advances in our understanding of the induced reprogramming of somatic cells to CMs. Starting from the growing understanding of heart development and from new insight in the epigenetic control of cardiac differentiation, we covered the progressions obtained in the cell culture approach and in the differentiation methods, the analysis of the secretoma of the hiPSCs differentiated cell, the new advances in hiPSC-derived bioengineered cardiac tissues, the exploitation of iPSCs-CMs for the in vitro modeling of cardiac diseases or for cardiac safety testing of drug and, finally, the immunological concerns associated with their clinical application.

## 2. Regulatory Pathways and Epigenetic Control of Cardiomyogenesis

Since hiPSCs can play a role in the therapeutic approach of CVD, a comprehensive understanding of the regulatory pathways that expand and functionally differentiate cardiac cells from their multipotent mesoderm precursors is required. Advances in cardiac progenitor cell biology are relevant, indeed, for the development of translation studies employing hiPSCs derived cells, since the possibility to obtain a near homogenous population of cardiac cells should help to minimize teratoma formation following cell transplantation.

The major steps of heart development are conserved between humans and other mammalians. This step by step complex (that have already been thoroughly reviewed in [9,10]) consists of a conserved regulatory network of transcription factors and signaling pathways that control specification, maturation, and maintenance of each of the multiple highly specialized myocardial lineages (ventricular, atrial, and conduction system cells).

### 2.1. Mesoderm Induction, Cardiac Specification, and Differentiation

During embryonic development, the formation of the nascent mesoderm requires the spatially and temporally regulated expression of Wnt, BMP, and Nodal/Activin pathway molecules. These factors regulate the entrance and the migration of the epithelial cells in the gastrulating epiblast, resulting in the generation of mesodermal cells marked by the expression of Brachiury T (Bry) [9].

Subsequent fate restriction of mesodermal precursors toward CV and hemopoietic progenitors can be identified by the expression of other specific factors. Indeed, the heart forms soon after gastrulation in the anterior mesoderm adjacent to the endoderm, whereas blood cells arise from the posterior mesoderm. Since bone morphogenetic proteins (BMPs) 2 and 4 are expressed in the lateral endoderm along the entire anterior-posterior axis, whereas heart induction is restricted to the anterior part, this implies that additional factors are required for the cardiac commitment of the undifferentiated mesodermal cells. One key gene in heart development is the mesoderm posterior 1 (Mesp1), which is considered the “master regulator” of cardiac progenitor specification [11,12,13]: in fact, it drives cardiac differentiation via the DKK1-mediated inhibition of Wnt signaling [13]. Mesp1 has been correlated with the definitive cardiac commitment by activating the expression of CV lineage defining transcription factors such as Nkx2.5, Isll, and myocardin. 

Once these myocardial precursors have fully committed, it is necessary to activate the Wnt pathway to facilitate the full maturation into differentiated CMs [9,11,13].

### 2.2. The Wnt Signaling

The neuronal tube and the adjacent notochord are potent sources of signals that repress cardiogenesis in the neighboring mesoderm. In particular, Wnt genes are highly expressed by the neuronal tube. Wnt proteins bind the frizzled receptors that block glycogen synthase kinase-3 (GSK3). This enzyme, when active, phosphorylates the β-catenin, resulting in its degradation by ubiquitin-mediated proteolysis. Thereby, Wnt signaling blocking GSK3 activity prevents the degradation of β-catenin that is able to move from the cytoplasm to the nucleus where it activates the Wnt target genes. In this way, Wnt signaling blocks cardiogenesis in the posterior mesoderm. On the other hand, Wnt signaling must be blocked to permit the heart development from the Mesp-1^+^ cells in the anterior mesoderm. Crescent is a family of proteins that share homology with the extracellular part of the Wnt receptor. Crescent is present in the anterior part of the mesoderm where another Wnt antagonist, DKK-1 is also expressed: in this way, the anterior mesoderm becomes permissive for heart formation interfering with the signal of Wnt [14,15].

### 2.3. Epigenetic Regulation of Human Cardiac Differentiation

During cardiac differentiation, cells express specific genes in a temporal and spatially accurate manner. The development of the mammalian heart is indeed dependent on the activation of a gene program regulated by specific histone modifications, nucleosome remodeling, and DNA methylations [16]. The epigenetic modifications that occur across the genome induce a chromatin pattern that is coordinated with the stage-specific expression of cardiac genes. This temporal evolution of histone modifications is a chromatin “signature” [17,18].

Table 1 summarizes the recent advances in epigenetic control of human cardiogenesis and cardiac differentiation. The histone modifications mainly include methylation or acetylation/deacetylation (by histone acetyltransferases or HAT and deacetylates or HDAC), whereas the DNA methylation involves the covalent transfer of a methyl group to the C-5 position of the cytosine ring by DNA methyltransferases (DNMTs). Short stretches of CG are often found at the gene promoter and their hypermethylation can facilitate the methyl binding domain association and the recruitment of chromatin remodelers for gene silencing and repressive histone modifiers [19]. The H3K4 methylation levels are fundamental for cardiac physiological function [18] as well as the removal of H3K29me3 activates the cardiac specific transcription factors Gata4, Nkx2.5, Srf and Tbx5 [20]. On the other hand, histone methyltransferases (HMTs) Smyd1 and WHSC1 are involved in CMs maturation [21]. Thompkins et al. [22] reported that DNMT1 expression decreases from cardiac mesoderm to CM stages, instead DNMT3A expression increases from ESC to primitive mesoderm stages.

Gilsback et al. [23] investigated DNA methylation in murine CMs and ES cells as a model for undifferentiated cell type. They showed that CMs have a short region of low DNA methylation in comparison with ES and these demethylated regions contained binding motifs for tissue-specific transcription factors. Some of the longest demethylated regions were identified in the cardiac ryanodine receptor (RYR2), titin (TTN), and in the α_1C_-subunit of the L-type Ca^2+^ channel.

Recently, it has been reported [24] that troponin T2 is highly expressed in CMs from fetal to adult stages and it shows sequential loss of CpG methylation (mCpG) and a promoter enrichment of active histone marks such as H3K9ac, H3K27ac, H3K4me3 and a genic enrichment of H3K36me3. Instead the Troponin I1 expressed at the fetal stage is silenced postnatally and, at the same time, a loss of both de novo mCpG and histone active marks occurs. Increase of expression of genes essential for myofibril (i.e., ACTN2, DES, CASQ2, MYH6, MYH7), sarcomere structure (i.e., ADRB1, HEY2, GATA4) and for regulation of the contraction (i.e., RYR2, S100A1, ATP2A2) is a consequence of the loss of mCpG. Changes in mCpG is accompanied by changes in histone marks. Demethylated region during maturation gained the active histone marks H3K27ac, H3K4me3, H3K36me3 and H3K9ac, whereas hypermethylated region showed a loss of these marks [24].

The role of epigenetic factors in controlling the cardiac lineage differentiation and specification is widely described and exploited to improve the cardiac differentiation of hiPSCs. 5-Azacytidine, an inhibitor of DNA methylation, promotes cardiac differentiation in ES and adult mesenchymal stem cells [25]. 

Recently, the long noncoding RNAs (lncRNAs), which are transcripts longer than 200 nucleotides that are not translated into protein, have gained widespread attention as potentially new and crucial regulators of cardiac differentiation [21]. In particular, lncRNA Braveheart seems to play a central role in cardiac differentiation, stimulating and maintaining CMs lineage commitment [21,26,27,28], while the lncRNA Fendrr, expressed specifically in embryonic lateral mesoderm, regulates heart development, most likely by modifying the chromatin signature of genes encoding transcription factors that direct cardiomyocyte differentiation [29].

The post-transcriptional regulation of the cardiac gene program also involves the microRNAs (miRNAs), non-coding RNAs of about 22 nucleotides in length that generally interact with the 3′ untranslated region (3′UTR) of mRNA target. This mode of pairing usually negatively regulates the translation of the target through the repression of the initial ribosome binding to the mRNA or the ribosome drop-off [21]. The analysis of miRNA expression in cardiomyocyte progenitor cells (CMPCs) showed that 188 miRNAs were detectable in proliferating CMPCs and 195 in differentiated CMPCs such as miR1, miR1-2, miR499, miR322, miR503, miR208, miR133, and miR26b [30,31,32,33,34]. MiR-208, together with miR-1, miR-133, and miR-206, are called myomiRs as they are expressed specifically in the heart and skeletal muscles. While miR-208 is expressed only in the heart, mir-206 is skeletal muscle specific [35]. Recently, the role of the miRNAs (in particular the let-7 family) during CM maturation has been also described [36].

### 2.4. The “Epigenetic Memory” in hiPSCs Differentiation Potential

Although hiPSCs can be generated from different somatic cells (fibroblasts, peripheral blood cells, keratinocytes), they maintain a residual DNA methylation signature transmitted from the parental cells, known as “epigenetic memory”, leading them to differentiate preferably into their original cell line [39,40,41]. Sanchez-Freire studied the contribution of epigenetic memory on the differentiation potential and maturity of hiPSCs derived from cardiac progenitor cells (CPC-hiPSCs) and dermal fibroblasts (Fib-hiPSCs). They found that Fib-hiPSCs had higher methylation levels of a region immediately upstream of the first coding exon of Nkx2.5 when compared to CPC-hiPSC. This evidence seems to suggest that the incomplete resetting of the pre-existent epigenetic state contributes to increased differentiation efficiencies and to the enriched cardiac gene expression observed in CPC-hiPSCs [42]. 

## 3. Generation of CMs from hiPSCs Culture

The number of protocols that derive CMs from hiPSCs have increased exponentially over the past decade and the differentiation protocols were modulated to generate mainly atrial-, ventricular-, and nodal-like CM subtypes. Important advances have been achieved in chemical-based cardiac differentiation, cardiac subtype specification, large-scale suspension culture differentiation, and the development of chemically defined culture conditions. These protocols of hiPSCs require key steps for the differentiation progression that have already been thoroughly reviewed [9,43,44].

In vitro differentiation of hiPSCs into CMs, regardless of the methodological approach, should mimic the sequential steps of in vivo embryonic cardiac development providing temporal administration of molecules that regulate specific signaling cascades: the activation of the canonical Wnt signaling induces the early primitive streak/mesoendoderm stage and the following inhibition of the same pathway at a later stage allows it to achieve the cardiac mesoderm specification [9,43,44].

Three main culture approaches have been described for small scale hiPSCs-CMs generation: (i) the co-culture of the hiPSCs with the inducing visceral endodermal cell line END-2. This was the first system used, but was also the least efficient one [10]; (ii) the embryoid body formation assay (EB) based on a three dimensional (3D) aggregation system; and finally (iii), the monolayer culture system used in many labs even if with different protocols. Large-scale cell cultures rely on culturing cells in dynamic suspension systems such as spinner flasks and bioreactors. A summary of the hiPSCs-CMs generation is reported in Table 2.

### 3.1. EB Formation-Based Differentiation Protocol

EBs are round, multi-cellular, 3D aggregates formed by hiPSCs and are able to differentiate into cells of all three germ layers including beating cardiomyocytes with low efficiency in the presence of fetal bovine serum [58,59]. In 2008, Yang et al. [45] established a three-step serum-free protocol characterized by subsequent supplementation of several cytokines (Activin A, BMP4, VEGF, DKK1, and bFGF) that resulted in a more efficient differentiation of the EBs into CMs. It became evident, however, that each hiPSC line needed optimal concentrations and timing of the administration of Activin A and BMP4, which are the factors responsible for the crucial step of mesodermal induction.

Recently, the discovery of the biological effects induced by the small molecules pushed scientists to apply them in stem cell biology. Karakikes et al. [46] increased the efficiency of beating iPSCs-CMs production by modifying Yang’s protocol [45] with the addition of the small molecules. Interestingly, the small molecule IWR-1, an inhibitor of the Wnt signaling pathway, caused all CMs to exhibit a typical ventricular-like phenotype, while the application of recombinant protein DKK1 generated a heterogeneous population that consisted of atrial-, ventricular-, and nodal-like phenotypes. 

However, the lack of uniformity in EB size, resulting in nonhomogeneous and asynchronous differentiation of the residing cells may hamper their effective employment in regenerative medicine. In 2007, Burridge et al. [60] modified the CMs differentiation protocol based on EB formation by introducing the forced aggregation technique, which was later improved using different engineered 2D and 3D technology to obtain controlled-size EBs [47,61].

More recently, Zhang et al. [48] combined the various specific advantages of existing protocols: EBs formation was performed by forced aggregation in serum-free medium supplemented with growth factors and small molecules. Interestingly, cardiac differentiation in the serum and serum/albumin-free basal media was improved by insulin supplementation during EBs formation, that resulted in 100% beating EBs, which were mostly ventricular or early ventricular-like cells. This differentiation method was easily translated to large scale CM differentiation by the generation of EBs and subsequent differentiation in static or dynamic suspensions [48].

### 3.2. Monolayer Culture-Based Differentiation Protocol

Although the 3D EBs format reproduces some aspects of the in vivo tissue architecture, the monolayer format is generally considered more reproducible and, in principle, a more suitable approach for the scale-up for clinical purposes. A 2D system guarantees, indeed, a more homogeneous exposure of the cultured cells to the soluble environment and might thus contribute to reducing the differences in the quality and quantity of CM differentiation between different cell lines. The first monolayer culture-based CMs differentiation of hESCs was performed by Laflamme et al. [20]. They cultured high-density undifferentiated hESCs as a confluent monolayer on Matrigel-coated plates and cells were sequentially treated with Activin A and BMP4; however, the differentiation efficiency was low and not successfully applicable to numerous other hESC or hiPSC lines. Improvements were then obtained with a Matrigel sandwich that facilitated epithelial–mesenchymal transition: layering on the cells a Matrigel overlay one day before the addition of the differentiation medium and maintaining it during induction with activin A, bFGF, and BMP4 resulted in CM differentiation efficiencies of up to 98% of cTnT positive cells [50].

Recently, Lian et al. [62] and Burridge et al. [52] looked into CMs with high efficiency (>95% cTnT Positive CMs). Both protocols are based on the application of two small molecules, a Gsk3- and a Wnt-inhibitor, that sequentially promote mesoderm formation and CM specification at precise developmental stages. However, unlike Lian’s observations, Burridge et al. [52] reported that albumin was necessary for CMs differentiation with high yield and purity. Differences between the two studies included the cell density at the beginning of the differentiation protocol and the concentration and the exposure windows for small molecules. Indeed, Lian et al. [62] reported that the optimal window for a Gsk3-and Wnt-inhibitor was from days 0 to 1 and from days 3 to 5, respectively, whereas Burridge et al. applied the Gsk3 inhibitor from days 0 to 2 and the Wnt inhibitor from days 2 to 4. Lian et al. also found that reducing the Gsk3-inhibitor concentration and/or treatment time in the absence of albumin permitted efficient mesoendoderm induction without cytotoxicity [21,22,23].

More recently, Parikh et al. [53] found that combining thyroid and glucocorticoid hormones during the cardiac differentiation process on a Matrigel mattress resulted in hiPSCs-CMs exhibiting T-tubule development, enhanced Ca-induced Ca release, and more ventricular-like excitation-contraction coupling.

Cao et al. [63] evidenced that hiPSCs cultured with small molecules in combination with growth factors induced the formation of multipotent cardiovascular progenitors that were able to stably self-renew and expand as a monolayer under feeder- and serum-free conditions. Most importantly, these CV progenitor cells retained the potential to efficiently generate CMs, smooth muscle cells, and endothelial cells in vitro [63]. The identification and isolation of a cardiac precursor cell population is expected to provide a source of cells for tissue regeneration, while also providing valuable insight into cardiac development.

### 3.3. Large-Scale CM Differentiation in Suspension Culture

10 billion hiPSC-CM is the number estimated for primate studies to be required for transplantation to restore function into infarcted human heart of a single patient. Development of robust, not expensive, and automated scalable suspension culture methods is required in order to generate large numbers of clinical grade CMs from hiPSCs. Up to date, several groups have focused on differentiation of the scale-up expanded hiPSCs to CMs by repeating already established differentiation protocols. Accordingly, several 3D suspension systems have produced CMs from hiPSCs by using matrix-dependent (microcarrier-based) and independent (microcarrier-free spheroid-based) systems. Niebruegge et al. [64] reported that inoculation of hESC size-controlled aggregates obtained combining suspension bioreactor and micro-contact printing steps led to the emergence of beating EBs after 2 weeks of culture and hypoxia further improved the efficiency of generated contracting EBs to approximately 50%. Encapsulated hESCs cultured in spinner flasks gave rise to more efficient CMs compared to their static culture [65].

Microcarriers promoted expansion of hESCs and hiPSCs in spinner flasks and controlled stirred tank bioreactors [54,55,56,57,58,59,60,61,62,63,64,65,66], while hydrogels have been used for developing a scalable 3D culture for hiPSCs expansion and cardiac differentiation [67].

In order to increase CMs production, Kempf et al. [56] have cultured hiPSCs as matrix-independent aggregates in a suspension culture which were directly differentiated to CMs according to Lian’s protocol [62], moving from a static system to rotating Erlenmeyer flasks, then to 100 mL stirred bioreactors using a cyclic perfusion feeding. The results indicated that the feeding strategy during expansion of hiPSCs resulted in the formation of approximately 470 μm hiPSCs aggregates that, once differentiated in the bioreactor toward the cardiac differentiation, generated 40 million predominantly ventricular-like CMs with up to 85% purity [56].

Darkins et al. have introduced a new approach for the design of large-scale manufacture of hiPSC-CMs that used biomechatronic methodology and computer-aided-design tools [68] in order to understand if certain configurations could be more favorable than others under given boundary conditions. They compared four different bioreactors with the tissue culture flask-based static conventional culture protocol to differentiate hiPSCs to CMs, considering several important parameters involved in large-scale manufacturing. The preferred reactor type was chosen according to a score matrix with the target specification attributes as discriminating criteria. They showed that stirred tank bioreactor with submerged culture had the highest score, followed by disposable wave bioreactor and rotating wall perfusion bioreactor. On the other hand, both the conventional protocol and hollow-fiber bioreactor had poor scores [68]. 

## 4. Morphological and Functional Properties of hiPSCs Derived CMs

Although it has been reported that in general, iPSCs-CMs structurally resemble embryonic or fetal CMs [10,69], it is well known that hiPSCs-CMs’ maturity depends on the time in culture. The hiPSCs-CMs phenotype, indeed, is strongly influenced by the timing of the culture and several groups have classified them as “early” and “late” hiPSCs-CMs. The early-phase characteristics are typical of the first month after starting the spontaneous beating whereas the late-phase characteristics develop afterwards [69,70,71].

Early phase hiPSCs-CMs (within 30–40 days post-induction) resemble embryonic or fetal mammalian CMs appearing as small (cell area: 400–500 μm^2^), rounded cells with some proliferative capacity lacking any discernible organized cardiac structure; immunocytochemical staining for α-actinin, a cardiac Z-disk protein, revealed poorly organized contractile machinery, characterized by low myofibril density and orientation, and variable Z-disc alignment [69,70,71]. They exhibit spontaneous contractile activity [72,73] and are characterized by a small negative membrane potential and small action potential amplitude [74,75]. Over the course of the next two months, hiPSCs-CMs lose their proliferative capacity [76] and change their morphology by becoming larger (cell area: 600–1700 μm^2^), more elongated, and with a lower circularity index. Like hESC-CMs, these late hiPSCs-CMs (between days 80 and 120 of in vitro development) demonstrate dramatic increases in the density and alignment of myofibrils throughout the cytoplasm and show repetitive banding patterns characteristic of organized sarcomeres with good registration across the entire width of the cell [69,70,71]. Different elements of maturity appear to be affected by hiPSCs line [77,78] or culture conditions [72,79]. However, late hiPSCs-CMs never reach either the dimension (cell area: around 1500 μm^2^) or the morphology of adult CMs, instead becoming closer to embryonic CMs. Indeed, adult CMs have elongated anisotropic shapes [80] and are aligned in the context of cardiac tissue. In vivo, immature CMs are rod-shaped, similar to the adult ones, but when cultured in vitro, the immature CMs flatten and spread in all directions while the adult ones maintain their cylindrical morphology in short term culture [81]. Thus far, hiPSCs-CMs have irregular shapes and they do not typically show alignment in two-dimensional cultures. These morphological differences are also reflected by a lower expression when compared to adult CMs of maturation-related sarcomeric genes such as *MYL2, MYH7, TCAP*, and *MYOM2,* and ion transport-related genes such as *KCNJ2* and *RYR2* [10,78,82,83,84,85]. Another aspect that confirms the immaturity of hiPSCs-CMs regards the localization of gap junction components. In adult CMs, these proteins accumulate at the intercalated disks, while in iPSCs-CMs, they are mainly localized at the circumference of the cell, recalling the structure of embryonic CMs [86].

The relative immaturity of hiPSCs-CMs also involves the development of the T-tubule network, a key component of excitation contraction coupling: extensive in adult CMs, it is absent in both iPSCs-CMs and embryonic CMs [87]. Since T-tubules allow an adult CM to have rapid electric excitation, initiation, and synchronous triggering of sarcoplasmic reticulum calcium release and, therefore, coordinated contraction throughout the entire cytoplasm, their lack of hiPSCs-CMs results in a lower excitation-contraction coupling, and in unsynchronized Ca^2+^ transients, as reflected by the non-uniform calcium dynamics across the cell and greater calcium peak amplitude in the sarcolemma than in the sarcoplasmic reticulum [88,89,90]. Thus, early iPSCs-CMs structurally resemble embryonic CMs, while late iPSCs-CMs develop a more adult-like morphology but do not appear to develop T-tubules.

Parikh et al. [53] broke the T-tubule barrier by discovering the appropriate combination of matrix and hormones to generate hiPSCs-CMs with a functional network of T-tubules producing more adult-like Ca^2+^ cycling. Their discovery of T-tubules in hiPSCs-CMs was a step forward, but the promise of adult-like hiPSCs-CMs in a dish has yet to be reached. The T-tubule network, in fact, lacked the abundance and detailed organization found in adult ventricular CMs and, although hiPSCs-CMs treated with T3 and Dex on the Matrigel mattress were larger cells, they were still smaller when compared to adult CMs.

Electrical immaturity of hPSCs-CMs is evident from spontaneous beating, since mature adult ventricular CMs are quiescent. Although the rate of contraction may be affected by cell line or culture conditions, the spontaneous and synchronous contraction of hiPSCs-CMs can be maintained over time in culture [91,92]. As reviewed by Denning et al. [93], the spontaneous beating depends on the high expression of the pacemaker current, I_f_, and low expression of inwardly rectifying potassium current, I_K1_, which stabilizes the resting membrane potential to around −85 mV in adult cells; in hiPSCs-CMs this value is −20 to −60 mV; density of I_Ks_ potassium and I_Na_ sodium channels is highly heterogeneous and can be lower than in adult. Collectively, these currents usually provide a capacitance of 30–50 pF versus ~ 150 pF in adult CMs and upstroke velocity of 10–50 V/s versus 150–350 V/s. The location of the gap junctions all around the cells instead of in the intercalated discs seems to be responsible for the slower conduction velocity in hPSCs-CMs (10–20 cm/s versus 60 cm/s). The differences in the physiological properties between adult- and hiPSC-derived CMs are summarized in Table 3.

Interestingly, hiPSCs-CMs differentiated from hiPSCs obtained from patients with long QT syndrome showed slower repolarization, thus recapitulating the in vivo behavior [94,95,96].

Common cardiac differentiation protocols produce predominantly ventricular cells with ~15–20% atrial cells and few nodal cells [97] as determined by electrophysiological analysis of action potential [9]. In clinical application, an enriched population of nodal-like cells could potentially be used in the formation of a biological pacemaker, whereas ventricular types may be used for recovery from myocardial infarction, or to evaluate drugs that that have *Torsade de Pointe* liabilities. It has been demonstrated that the pharmacological inhibition of NRG-1β/ErbB signaling enhanced the population of nodal-like CMs [98] and that retinoic acid could increase the proportion of atrial-like CMs whereas its inhibition could increase the proportion of ventricular-like cells [99]. Furthermore, it was possible to strongly increase the nodal population by inhibiting the neuregulin signaling using small molecules [100].

hiPSCs-CMs present cardiac specific inotropic and chronotropic receptors, other than the β1 and β2 adrenoceptor response [70,101,102,103]. Similar to adult CMs, isoprenaline increases both the contraction rate and the amplitude of the calcium transient, and decreases the relaxation time [102]; on the other hand, the observation that, unlike adult CMs, isoprenaline does not affect the contraction force [103] supports the functional immaturity of this cell type. Ravenscroft et al. [104] evidenced that CM microtissue co-cultured with cardiac endothelial cells and fibroblasts is superior in predicting inotropic responses than single-cell type CM microtissue.

## 5. hiPSC Paracrine Effects for Cardiac Repair and Regeneration

Despite the heart has always been considered as devoid of any regenerative potential, recent work has demonstrated that it is endowed with an endogenous restorative program based on the re-establishment of the modulatory activity of cardiac progenitor cells [100,105] along with resident cardiomyocyte proliferation [75]. While broadly active during developmental cardiogenesis and in the very early post-natal life, these mechanisms become quiescent and unresponsive soon after birth, leaving the heart with limited repair potential in pathological situations. Therefore, a working strategy is urgently needed to restore the potential for both cardiac repair and regeneration.

In this scenario, growing interest has been driven to the so called “stem cell-derived paracrine effect” as a putative working strategy to restore such dormant mechanisms of cardiac restoration. Indeed, it is now well accepted that either autologous or allogeneic transplantation of different populations of stem cells into the injured heart results in quite limited differentiation, while providing overall significant improvement in heart function [106]. Thus, the beneficial effects obtained following an injection of stem cells seem to be mainly due to their modulatory paracrine effects [107]. As a matter of fact, several studies have supported the paracrine hypothesis by reporting successful reduction of infarct size and improvement of angiogenesis and cardiac output that are most likely attributable to the release of soluble factors, rather than de novo cardiomyogenesis by the engrafted cells. Hence, the detailed analysis of the stem cell “*secretome*”—as the whole of growth factors and chemo-attractant molecules produced by paracrine secretion—has gained growing attention and the quest is now to identify the most suitable cell candidate to obtain the ideal paracrine cocktail of factors to be delivered to the injured myocardium. Several populations of stem cells have been evaluated, with adult somatic mesenchymal stromal stem cells (MSC) isolated from different tissues being the most investigated. However, while adult MSC may represent a feasible option given the ease of their collection from clinical waste material, (i.e., adipose tissue harvested during surgical procedure) they present several limitations to their therapeutic application such as low yield, invasive sampling, and controversial self-renewal. In contrast, hiPSCs may offer a valuable choice to overcome these limits given their pluripotency, high self-renewal, and embryonic stem cell-like properties. Most studies involving the use of hiPSCs for in vivo cardiac regeneration have focused on the exploitation of their cardiomyogenic differentiation potential. Nonetheless, hiPSCs have also been recently described as playing a significant role in modulating the cardiac microenvironment by mediating pro-survival effects while improving cardiac function and homeostasis via secretory mechanisms of action, thus suggesting a remarkable paracrine potential. Indeed, recent work from Yan and Singla [108] has shown that hiPSCs systemically transplanted into a preclinical mouse model of diabetes-induced cardiomyopathy contributed to the increase in antioxidant levels, and counteracted adverse cardiac remodeling while improving cardiac function by acting on the Akt, ERK1/2, and MMP-9 signaling pathways via multiple paracrine mechanisms. Likewise, when considering a chemotherapy drug-derived murine model of cardiomyopathy, as the one obtained by regular administration of the oncological drug doxorubicin, a well-known cardiotoxic agent, hiPSCs transplantation following ischemic injury resulted in the decrease of cardiac apoptosis and interstitial fibrosis via paracrine modulation of Notch-1 signaling [109]. Further studies have highlighted the potent paracrine effect of the secretome of hiPSC-derived MSC as specifically enriched with macrophage migration inhibitory factor (MIF) and growth differentiation factor-15 (GDF-15) citokines; indeed, the iPSC-derived-MSC-conditioned medium was shown to exert remarkable cardioprotective effects on neonatal rat CMs and murine cardiac tissue against anthracycline-induced cardiomyopathy [110]. 

In another study, [111], the hiPSCs secretome has been used to prime endogenous progenitor cells such as cardiac mesenchymal stromal cells (cMSCs) and evaluate changes in their proliferative, survival, and differentiation potential. In particular, human hiPSCs-secreted extracellular vesicles/microvesicles (hiPSC-MVs) proved successful in mediating the transfer of their bioactive cargo (mRNA, microRNA, and proteins) to the target cMSCs culture. Most importantly, hiPSC-MVs modified the transcriptome and proteomic profiles of target cells, triggered changes in their metabolism, cell cycle, other than increasing the proliferative and anti-apoptotic effects on cMSCs. Overall, this secretome pushed the target cMSCs to a more primitive state, enhancing their cardiac and endothelial differentiation potential, thus further supporting the promising therapeutic potential of this approach [111].

In the last few years, we have witnessed the dramatic and rapid expansion of hiPSCs biology and preclinical application of hiPSCs-cell derivatives for future therapy. Nonetheless, several reports have indicated limited engraftment of hiPSCs-CMs when transplanted in vivo; yet, there is evidence of improvement of resident cell survival and local angiogenesis along with remarkable decrease of fibrosis and inflammation, following injury. These results are likely to be due to paracrine modulatory effects exerted by the transplanted hiPSCs-CMs on the neighboring resident cells via the secretion of biologically active extracellular vesicles including exosomes [112]. Indeed, immunosuppressed mice experiencing a myocardial infarction showed better outcomes when transplanted with hiPSCs-CMs when compared to undifferentiated cells, despite the poor cell engraftment in both treated groups, suggesting differential paracrine effects, with differentiated cardiac lineage cells contributing more significantly via the secretion of promigratory, proangiogenic, and antiapoptotic mediators [113].

In light of such evidence, hiPSCs can offer an appealing therapeutic tool for future cardiac regenerative medicine via the combined advantage of their pluripotency and peculiar paracrine potential.

## 6. Advanced Technologies and Tissue Engineering: Novel Approaches for Studying hiPSC-Derived Cardiac Tissues

In parallel with the revolution brought by the use of hiPSCs, the advances in cell culture techniques and methods have led to more innovative approaches to personalized medicine. Engineering approaches to stem cell cultures can open doors into previously inaccessible scenarios and poorly understood biological phenomena. Although still key for biological discoveries, standard culture techniques in Petri dishes cannot be fully representative of the mammalian in vivo complexity. This is due to a series of limitations such as: (i) 2-dimensional (2D) growth; (ii) poor mimic of in vivo structure and substrate compliance; and (iii) batch-wise operations (media change, addition of drugs or other factors, etc.) that result in unpredictable kinetics, poorly defined timescales, and the lack of precise patterns of stimulation [114]. In contrast, advanced “bioreactor-based” culture techniques have overcome such limitations and offer a series of main undeniable advantages [115]. Focusing on the microscale, on devices with reduced size and features ranging from a few microns to a few centimeters, we gain: (i) feasibility of working in 3D; (ii) better mimic of the in vivo microenvironment (we are closer to the characteristic sizes of cells and extracellular structures); (iii) steady state conditions, translating into operating parameters that are constant in time and are kept at well-defined values, and in the possibility of introducing precise spatial and temporal patterns of stimulation; and (iv) increased throughput [116,117]. Focusing on transport phenomena, when using dynamic systems (where culture media flows through the device) we can control both convection and diffusion, thus enabling the generation of complex patterns of stimulation on the cellular microenvironment. The microscale here plays again to our advantage, as the typical length scales and flow rates determine the establishment of a laminar regime, one where velocity and concentration profiles are more easily controlled and modeled/predicted [118]. Advanced culture systems are also more amenable to integration with sensing elements for online measurements and monitoring of the culture maturation [119]. Finally, a downsizing of culture systems results in reduced costs and the number of cells and reagents used, an important advantage especially for experiments involving hiPSCs and hiPSCs-CMs.

Nowadays, heart-on-chip technologies are developing at a fast pace thanks to the strong drive towards improving the drug development process and reducing multi-billion USD losses due to late stage failures (reviewed in Conant [120]). Novel technological approaches involving heart cells/tissues are fundamental for two main reasons: (i) the need for new and improved drugs for heart conditions and; (ii) the need for earlier and more effective evaluations of cardiotoxicity of other drugs.

The promise of using hiPSCs-CMs for transplantation to infarcted hearts has not been entirely fulfilled, with one of the biggest limitation being the mismatch between their electrophysiological properties and those of the native tissue. As described above, hiPSC-CMs typically present immature phenotypes, characterized by sarcomeres lacking H zones, I bands and M lines, by poorly controlled spontaneous beating, deregulated action potentials, altered calcium handling properties and incomplete connexins-mediated coupling when interfaced with the host environment. Ravenscroft and colleagues tested a coculture tissue engineering approach, based on the hypothesis that the presence of non-myocyte cells will promote CMs maturity, and demonstrated that CMs microtissue cocultured with cardiac endothelial cells and fibroblasts is superior in predicting inotropic responses than single-cell type CMs microtissue [104]. Recently, Pallotta et al. [121] proposed a model of bioengineered cardiac tissues preconditioned with BMPs—proteins usually secreted by macrophages present at the site of myocardial infarction—that improved CMs functionality, cardiac gene expression and the ability to sustain angiogenesis in vitro based on diffusion of the exogenous BMPs. This model might represent a step towards the validation of more complex bioengineered constructs, in which protein diffusion and degradation rate of the biomaterial can be tuned to achieve a suitable protein release for in vivo applications

Precise engineering of the culture systems allows for the introduction of physiologically relevant stimulations such as spatial (i.e., 3D culture), topographical, electrical, and mechanical. In particular, electrical stimulation—a known strong effector for cardiomyocytes maturation—has often been used in conjunction with advanced technological solutions to push cell maturation [122,123]. Biowires™, as an example, derive from the combination of hiPSCs-CMs, 3D cell cultivation systems, and electrical stimulation specifically tailored to generate tissues with more mature structural and electrophysiological properties [124]. Biowires™ present improved ultrastructure organization with, among others, clearly visible Z discs, H zones and I bands, correlated with lower excitation threshold, higher conduction velocity, and improved Ca^2+^ handling properties. Recently, Ronaldson-Bouchard and colleagues [125] have demonstrated that adult-like human cardiac tissue can be grown from hiPSCs-CMs in fibrin hydrogel subjected to stretch and auxotonic contractions in just four weeks of in vitro culture. Two methodological advances underlie the accelerated cardiac maturation: the formation of tissues from early-stage hiPS-CMs, which displayed marked plasticity immediately after the initiation of spontaneous contractions; and physical conditioning with increasing intensity. Tissues showed electrophysiological properties that were comparable to Biowires, including the shape of the action potential with its characteristic notch, the resting membrane potential, the *I*K1 current and the conduction velocity. Moreover, tissues were characterized by adult-like gene expression, oxidative metabolism, positive forced frequency relationship and physiological calcium handling.

Bursac’s group [126] faced another fundamental challenge obtaining sufficient numbers of hiPSC-CMs for regenerative medicine applications (over 1 billion cells). They developed “cardiobundles” and a “cardiopatch” platform for the 3D culture and maturation of hiPSC-CMs over a period of 5 weeks. The platform succeeded in producing constructs that showed robust electromechanical coupling, consistent H-zones, I-bands, and evidence for T-tubules and M-bands. The cardiopatches could be scaled up to clinically relevant sizes while maintaining their physiological properties.

**Figure 1:**
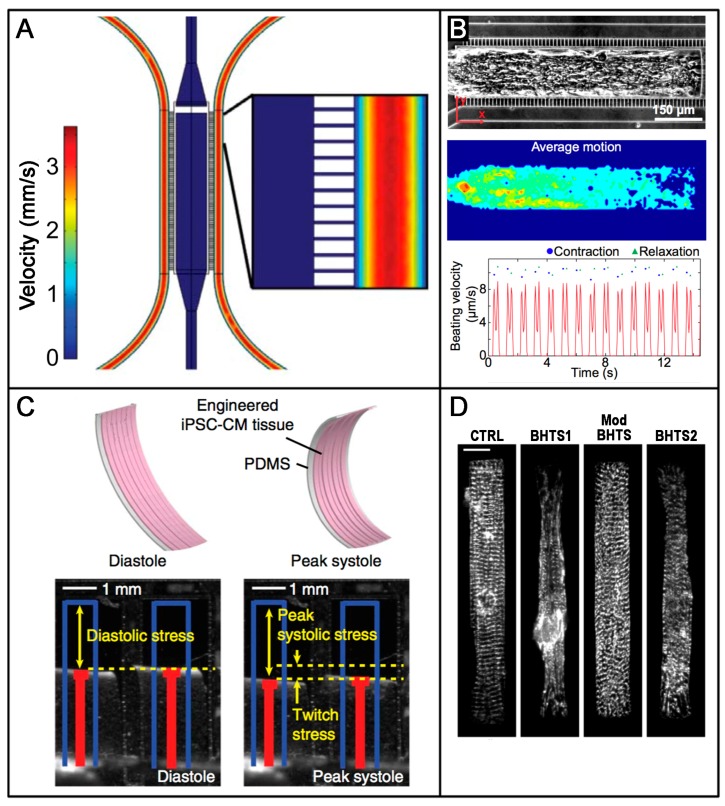
(**A**). Schematic of the device: the central cell loading channel is connected to the lateral C-shaped medium-delivering channels by a “ladder” of thin microchannels, purposely design to protect cells from shear and switch to a diffusive mass transport regime. The colors are representative of the linear velocities on a chosen plane, and show decreasing values from red to blue; (**B**). Characterization of the 3D cardiac tissue formed in the middle channel. Top: optical microscopy image showing tissue density and overall organization and alignment; middle: heat map of the average motion generated by the contractile activity and, bottom: corresponding average beating kinetics. Adapted with permission from Mathur et al. [127]; (**C**). Top: schematic representation of contracting constructs and approach to measurements; Bottom: iPSC-CMs seeded onto thin elastomers with patterned lines of fibronectin self-organized into microscaled myocardial tissues and exhibited contractile properties in response to electrical stimulation; (**D**). Representative images showing actinin staining of iPSC-CMs on micropatterned fibronectin rectangles. BTHS iPSC-CM micro-tissues show impaired sarcomere organization (BTHS1 and 2 in Galactose and Glucose medium, respectively), while cells transfected with TAZ modRNA (Mod BHTS) clearly demonstrate a rescued organization, comparable to that of the control cultures (CTRL). Adapted from Wang et al. [128].

Among others, Healy’s group [127] developed an interesting microphysiological system (MPS) that integrated the use of hiPSC-derived CMs with advanced microfluidic platforms that has proved useful in pharmacological studies. Their design ensured that cells self-organized into an aligned 3D micro-tissue and enabled the generation of tissue-like gradients of drugs in a shear-protective environment (Figure 1A), mimicking that provided by the endothelial barrier. hiPSCs-derived CMs organized and started to spontaneously contract within seven days of culture, and their movement could be tracked and measured online with non-destructive imaging techniques (Figure 1B). The authors successfully used their MPS to test the cardiac response of four model drugs and obtained data showing half maximal inhibitory/effective concentration values that were more representative of whole organ responses than those typically obtained at the cellular scale. Overall, their results suggested how these approaches could significantly improve the outcomes of in vitro screening studies of drug efficacy and cardiotoxicity.

Great efforts are also being devoted towards the development of heart-on-chip technologies to model human disease. In a relevant example of this matter, Wang et al. [128] used advanced technologies to gain insight into the pathophysiology underlying the frequent cardiomyopathy experienced by patients affected by Barth syndrome (BTHS), a mitochondrial disorder. Their bioengineered microchips were based on thin elastomer films that functionalized with micropatterned thin strips of fibronectin (rectangles ~100 × 15 μm length × width, and lines 15 × 2 μm width × spacing). BTHS hiPSCs-CMs, when seeded on top of these films were organized following the pattern dictated by the adsorbed protein, generating a laminar anisotropic myocardium. Their dual approach allowed: (i) the quantification of the contractile properties of the engineered myocardial tissues (Figure 1C); and (ii) the evaluation of the phenotypic maturation of the constructs based on the analysis of sarcomeres and fibrous structures organization (Figure 1D). The main results proved that: (i) engineered BTHS hiPSCs-CMs micro-tissues exhibited impaired sarcomere assembly; and (ii) they correctly recapitulated the pathophysiology of BTHS cardiomyopathy by developing significantly lower twitch and peak systolic stress, both when compared to the controls. Finally, and most importantly, the authors proved that engineered tissues effectively modeled disease correction showing restored contractile function after treatment with TAZ modRNA.

In a concerted effort, a consortium led by top scientists across the US is actively working to develop an integrated microphysiological platform, HeLiVa, capable of reproducing the complexity of the “whole body” [129]. HeLiVa is an integrated heart–liver–vascular system for drug testing in human health and diseased settings. The micro-tissues are produced starting from a single line of human pluripotent stem cells (and are thus patient-specific), and the platforms are compatible with real-time biological readouts. Once again, the technology-enabled production of functional human tissue units and their use in studies seeking to measure physiological responses to known or pipeline drugs, greatly benefit from their higher biological fidelity and can be transformative to drug screening and the modeling of disease.

In recent years, there has also been a great drive towards the birth of start-ups and university spin-offs devoted to the fast translation of laboratory-scale discoveries to their wider adoption and application. An example is Novoheart (www.novoheart.com), a company offering customized screening and phenotyping services on hESC-derived ventricular cardiomyocytes based on their proprietary MyHeart™ platform. The platform can be adapted to single cells, anisotropic sheets, and tissue strips. Their human ventricular cardiac organoid chambers provide a comprehensive bioreactor model enabling force and electrophysiology measurements with minimal manipulation [130].

## 7. The Promise of hiPSCs-CMs in Biomedical Applications

hiPSCs demonstrate pluripotency, the ability to self-renew and are patient-specific. Based on these features, hiPSCs are expected to be applicable in drug discovery, disease modeling, and cell therapy.

***Screening for drug discovery and cardiotoxicity testing.*** Safety in pharmacology is important as the heart is sensitive to the side effects of drugs. In fact, drugs that cause heart damage as a side effect have been implicated in around 30% of drug withdrawals in the US over the past 30 years [44]. The relative ease of efficient reprogramming and directed cardiogenesis has accelerated progress towards biomedical application, with particular attention to drug screening. This is helped by hiPSCs largely eliminating ethical or legal restriction that prohibited use of hESC in many companies or countries. Drug-induced cardiotoxicity can adversely affect myocardial contractility through structural (non-proarrhythmic) or electrophysiological (proarrhythmic) changes in CMs, either of which can result in loss of contractility and cardiac function. hiPSCs-CMs are a promising human cardiac in vitro model system to assess both proarrhythmic and non-proarrhythmic cardiotoxicity of new drug candidates and published studies have demonstrated that they hold great promise in cardiac safety testing [131]. Current in vitro cardiac models for contractility include primary cultures of adult human CMs, isolated Langendorff heart and primary cultures of neonatal mouse or rat CMs. One big challenge to the use of primary cultured CMs is that this in vitro model does not remain viable for long-term culturing; moreover, it is known that acutely isolated CMs are quickly overrun with fibroblasts. Because of this culture limitation, most primary CMs experiments are designed with a short drug exposure time (a few minutes to a few days). For drugs given to humans on a long-term basis, such as kinase inhibitors, acute exposure in vitro studies may not detect effects related to long-term structural damage. In contrast, the Langendorff heart model keeps the intact function of the working myocardium and the coronary vessels following longer-term dosing in animals and therefore a variety of functional parameters can be measured within one single heart. However, this model is somewhat difficult to perform routinely and does not address the concerns for detecting human-specific responses. On the other hand, the hiPSCs represent a renewable cell source of CMs and overcome species differences present in animal models allowing a human-specific assessment of drug-induced contractility changes: expressing human cardiac ion channel, hiPSC-CMs respond to major channel blockers [132] and can also be useful to detect drug effects mediated by cell surface receptor binding [133] mitochondrial damage [134], oxidative stress [135], Ca^2+^ handling [136,137], or intracellular messengers [138]. 

Recently, Sharma et al. [136] used hiPSC-CMs to screen FDA-approved Tyrosine kinase inhibitors (TKIs) by measuring alterations in cardiomyocyte viability, contractility, electrophysiology, calcium handling, and signaling. With these data, they generated a “cardiac safety index” to assess cardiotoxicities of existing TKIs (Table 4).

Burridge et al. [134] showed that patient-specific hiPSCs-CMs can recapitulate individual propensities to doxorubicin-induced cardiotoxicity.

The Comprehensive in vitro Proarrhythmia Assay (CiPA) initiative represents a paradigm shift for proarrhythmic risk assessment, and, recently, hiPSCs-CMs have been proposed in the CiPA scheme to improve the non-clinical evaluation of proarrhythmic liabilities of new drugs [139]. Yang and Papoian [140] have recently suggested to complement the electrophysiological evaluation of the CiPA platform by adding a structural assessment of hiPSCs-CMs, thus, allowing to analyze both potential proarrhythmic and non-proarrhythmic effects on cardiac contractility. Even if the in vitro screening assays for cardiotoxicity are predominately focused on identifying proarrhythmia risk, some platforms have already been developed to detect both proarrhythmic and structural toxicities [141]. Importantly, this combined approach provide an integrated structural and electrophysiological assessment in CMs that might better predict the drug-induced contractility changes.

In Japan, the Consortium for Safety Assessment using hiPSCs HEART team has been working on hiPSCs-CMs in the Multi-electrode array (hiPSCs-CMs/MEA) under a standardized protocol for proarrhythmic risk assessment. Recently, Nozaki et al. [142] evaluated the responses of hiPSCs-CMs/MEA to 31 compounds of different categories (ERG channel blocker or activator, late Na current inhibitors and enhancer, Ca channel activator, multi-ion channel blockers, etc.) associated with cardiac toxicities, demonstrating that the hiPS-CMs/MEA assay might constitute a core platform for cardiac safety assessment where drug-induced arrhythmogenesis might be evaluated using hiPSC-CMs under non-clinical setting. This report would provide CiPA with informative guidance on the use of the hiPSCs-CMs/MEA assay, and promote the establishment of a new paradigm, beyond conventional in vitro and in vivo assays for cardiac safety assessment of new drugs.

As each cell culture model, hiPSCs-CMs present some limitations which may impact their utility in safety assessment. A major criticism of hiPSC-CMs is their immature phenotype when compared to adult CMs [71,93]. The immaturity is reflected in a less-negative resting membrane potential, lack of T-tubules and fetal-like morphological parameters. Because of this immaturity, hiPSC-CMs display a different contractile response to inotropic compounds when compared to adult cells: hiPSC-CMs are indeed unable to generate a positive inotropic response to isoproterenol treatment and display a negative force–frequency relationship [143].

Finally, the discover that the miRNA profile of hiPSC-CMs is affected during early and late stages of treatment with the cardiotoxic drug doxorubicin [144] might lead the discovery of novel class of biomarkers, useful to monitor potential drug-induced cardiotoxicity in patients before irreversible cardiac damage has occurred.

***CV disease modeling***. Since hiPSCs generated from individuals with genetic disorders maintain the anomalies [145], patient-derived hiPSCs are candidates to model the molecular basis of pathologies and to investigate their phenotypes, the disease mechanisms and the drug response/toxicity. Recently, hiPSCs have been modeled for several cardiac pathologies including familial dilated cardiomyopathy [146], Barth syndrome [128], arrhythmias (LQT1, LQT2, LQT3, and LQT8/Timothy syndrome) [40,94], catecholaminergic polymorphic ventricular tachycardia [40,147], and LEOPARD syndrome [148].

***Cell therapy and myocardial infarction repair****.* hiPSCs-CMs are of great interest for cell-based heart regeneration. To avoid arrhythmia, which is the most severe side-effect of cell replacement therapy, it is essential to implant relatively homogeneous, probably mature CMs that have ventricular phenotypes. Several groups have reprogrammed murine embryonic fibroblasts to iPSCs, that when injected intramyocardially in immunodeficient mice after coronary artery ligation were able to differentiate into CMs, vascular smooth muscle cells, and endothelial cells determining an improvement of the ventricular function. However, the observation that transplanted iPSCs could generate tumors in the recipient mice strongly limited the possibility of translating these research findings into clinical practice [7,149]. Following studies focused on transplanting iPSC-derived cardiac progenitors cells [63] or iPSC-CMs [150] into the infarcted area of immunocompetent mice, the data showed an improvement of ventricular contractility without tumor formation.

The main limitation of regenerative medicine is the poor cell retention into the organ after injection, with 95–99% of grafted cells lost within a few days. Cell retention can be enhanced by delivering cells on biomaterials such as hydrogels, tissue patches, or scaffolds [67]. Myocardial tissue engineering structures may be porous or dense (patches), depending on the purpose of the construct. If the engineered biomaterial is to support and possibly remold the infarcted area over a period of time, then it is that vital the construct is a scaffold that consists of interconnected pores (>90% porosity) with diameters ranging between 300 and 500 µm for cell survival. This will allow cells to exchange nutrients and remove cellular secretions, enhance cell penetration and tissue vascularization. On the other hand, if the biomaterial will serve solely as a means of cell transport, to deliver cells to the desired region only and degrade over a given period of time (e.g., within 3 months), a dense patch will be adequate for this purpose. Both these approaches can reduce mechanical stem loss and provide a protective environment for cell survival [151]. Considering that growth factors are important signaling molecules in the control of tissue regeneration, the application of growth factors within biomaterials in tissue engineering represents a powerful tool for controlling cell survival and differentiation.

Recently, Menaschè et al. [152] showed the feasibility of generating a clinical-grade population of human ESC-derived cardiac progenitors on the first clinical case report. These cells were combined within a tissue-engineered (a fibrin scaffold) construct and then transplanted in patients with ischemia-induced heart failure [153]: of the six enrolled patients, one died early post-operatively for treatment-unrelated comorbidities while all others had uneventful recoveries. None of the patients developed tumors or presented arrhythmias. Three patients developed clinically silent alloimmunization. All patients were symptomatically improved, one patient died of heart failure after 22 months. This trial demonstrates the short- and medium-term safety of hESC derived cardiac progenitors. Considering the similarity between hESCs-CMs and hiPSCs-CMs, this result opens new opportunities for the clinical application of hiPSCs.

## 8. The Immunological Challenges of hiPSCs and the Generation of Haplobank for the Cell Therapy

To date, it is still unclear how the immune system of potential recipients might perceive tissues differentiated from hiPSCs (reviewed in [154]). Some studies predicted that iPSCs-derived tissues may not be immunogenic since, unlike solid organs from living or cadaveric donors, tissues from hiPSCs lack both the endogenous dendritic cells and the lymphatic drainage required for the emigration of the immunological cells into the secondary lymphoid tissues of the recipient; other studies have highlighted the capacity of hiPSCs to actively exert a local nonspecific suppressive effect on T cells [155,156,157]. Interestingly, some authors suggested that the epigenetic memory of the hiPSCs could influence the immune response elicited by their administration. Wang et al. [158] demonstrated that the iPSCs lines derived from Sertoli cells of the mouse testis, an immunological-privileged site, were significantly less immunogenic when transplanted into allogeneic recipients than iPSCs derived from fibroblasts. Thus, in addition to the intrinsic immunosuppressive features of iPSCs, the epigenetic characteristics associated with immune privilege may also be exploited to reinforce the iPSCs’ capacity to evade immune recognition. Unfortunately, the immunological consequences of iPSCs transplantation are still uncertain and affect the planning of clinical trials. Indeed, although iPSCs seem to be immunologically privileged, evidence suggests that the immune response of the recipient may oppose the engraftment and-/or the persistence of the iPSCs. It has been reported that iPSCs lines transplanted in syngeneic recipients attracted a significant T-cell infiltrate that led to their rejection [159]. The extent of such immunogenicity appeared to decrease with differentiation, but some terminally differentiated cell types, such as smooth muscle cells, retained significant immunogenicity that led to their demise upon transplantation [159,160].

The immune response evoked by transplanted iPSCs may be ascribed to the ectopic expression of “developmental antigens” [154]. Pluripotency-associated genes are expressed at high levels within the early embryo and are normally downregulated upon implantation, being extinguished long before thymic development and selection of the T-cell repertoire. Reprogramming of adult somatic cells to pluripotency is responsible for the re-expression of developmental antigens, which are not properly downregulated during differentiation in vitro, most likely accounting for their rejection, even by autologous recipients. 

hiPSCs technology supports also the possibility to establish a “stem cell haplobank” in order to facilitate rudimentary matching with potential recipients. The opportunity to pre-select donors with a desirable haplotype for the generation of hiPSC lines opens up an opportunity, not feasible with hESC, to create a bank of cell lines specifically chosen to match the widest possible number of recipients worldwide [161]. This shall be done by recruiting donors who are blood group O and are homozygous for common human leukocyte antigens (HLA). The haplobank would allow to match hiPSCs and recipient for selected HLA loci (in particular HLA-A, -B and -DR), as well as in solid organ transplantation. In any case, it should be taken into consideration that different HLA loci and minor histocompatibility antigens might be responsible of rejection. This event is generally prevented by a long-term immunosuppressive therapy, that should be discouraged with hiPSCs transplantation, because of the recognized risks of tumorigenesis of these cells. Consequently, the success of an hiPSC haplobank is linked with the development of alternative strategies for immune intervention. While studies have proposed the encapsulation of the hiPSC-derived tissues in order to provide a protective barrier against the recipient’s immune system, other recent studies have suggested more subtle opportunities for immune intervention and for inducing the tolerance state [159,160,161]. Although the challenge remains considerable, evidence indicates that the induction of immunological tolerance to hiPSC-derived tissues is feasible and significantly easier than the establishment of tolerance to tissues from conventional sources. In particular, the microenvironment created by hiPSC-derived tissues supports the establishment and maintenance of tolerance, suggesting that such grafts may actively participate in their own survival.

## 9. Conclusions

hiPSCs-CMs are a promising tool in drug discovery, disease modeling, and cell therapy, but despite the high hopes and expectancy, issues with the reprogramming technology and the biology of reprogrammed cells still cast a shadow on the clinical application of hiPSCs. The technical hurdles in reprogramming have resulted in diversity in the quality of hiPSCs generated, the “epigenetic memory” influences the differentiation efficiency, and the reprogrammed cells present poorly controlled risks of unpredictable reactions in both the processes of dedifferentiation and subsequent differentiation of the cell strains employed for therapeutic or experimentation goals.

However, although the reprogramming technology that creates hiPSCs-CMs is currently imperfect and much additional basic research will be required before its clinical application, these cells will likely impact future therapy, representing multi-purpose tools for medical research and illuminating many areas related to CV disease. Use of patient-specific hiPSCs-CMs may mirror clinical outcomes of drug-induced cardiotoxicity [134,162], and may be used for drug screening in the future. For precision medicine, creating biobanks that include both diseased hiPSCs and genetically matched controls has been proposed as a useful resource to study interpatient variation and changes in metabolic and stress-response genes that help risk-stratify patient-specific susceptibility to drug-induced cardiotoxicity [163].

## Figures and Tables

**Table 1:** Recent advances in epigenetic control of human cardiogenesis and cardiac differentiation.

Epigenetic Modifications	Name	Action	Reference
***Histone acetylation***	Histone acetyltransferase (HAT)	P300 is essential for cardiac development. It contributes to Gata4, Srf, Mef5c expression. P300 knockout mice are embryonically lethal	[37]
	Histone deacetylase (HDAC)	Mice lacking both HDAC1 and HDAC2 show neonatal lethality due to arrhythmias and dilated cardiomyopathy	[38]
	Inhibitors of HDAC	Trichostatin A promotes cardiac differentiation increasing expression of Gata4, Mef2c and Nkx2.5	[25]
	H3K9ac and H3K27ac	In CMs from fetal to adult stages, TNNT2 shows a sequential enrichment of active histone markers such as H3K9ac and H3K27ac	[24]
***Histone methylation***	Histone methyltransferases (HTMs)	Loss of HMT Smyd1 is embryonic lethal, because mice show right ventricular hypoplasia and impaired cardiomyocyte maturation.	[21]
		HTM WHSC1 is involved in Nkx2.5 repression via H3K3me37.	
	Histone demethylase (HDMs)	The HDM UTX removes H3K29me3 activating the cardiac transcription factors Gata4, Nkx2.5, Srf, Tbx5. Mice lacking UTX show severe heart malformation.	[20]
	H3K4me and H3K27	H3K4 methylation levels are fundamental in murine CMs. A loss of H3K4 methylation can result in intracellular calcium modifications and increased contractility	[18]
		FGF19 and NODAL genes show high levels of H3K4me3 and H3K27me3 in undifferentiated ESC and low levels during the differentiation	[17]
		Cardiac transcription factorsGata4, Wnt2, Tbx2, Nkx2.5 show high levels H3K27me3 during the pluripotency that decrease during differentiation, in the same time there is a gradual increase in H3K36me3 and H3K4me3	
		Wnt, Hedgehog, TGFβ family, VEGF, FGF family, PDGF(pathways involved in cardiac differentiation) show a stage-specific repression by H3K27me3 and activation by H3K36me3 and H3K4me3	
***DNA methylation***	DNA methyl transferase (DNM)	DNMT1 expression decreases from mesoderm to CM stage while DNMT3A increases from ESC to primitive mesoderm stage. WNT and TGF-β genes undergo promoter methylation changes, the latter pathway became hypomethylated and upregulated in CM stage, whereas generally WNT genes acquire promoter methylation	[22]
	Inhibitors of DNA methylation	5-Azacytidine promotes cardiac differentiation in ES and adult mesenchymal stem cells	[25]
	mCpG	In CMs from fetal to adult stages, TNNT2 shows a sequential loss of mCpG, instead fetally expressed TNNI1 is silenced postnatally and there is a loss of de novo mCPG.	[24]
		Comparison of mCpG changes during development of fetal and maturation of infantile CMs showed a predominant loss of mCpG	
		Changes in mCpG is accompanied by changes in histone marks. Demethylated region during maturation gained the active histone marks H3K27ac, H3K4me3, H3K36me3 and H3K9ac, whereas hypermethylated region showed a loss of these marks	
***Long non*** ***-*** ***coding RNA***	Braveheart	Braveheart is an activator of Mesp1, Gata4, Nkx2,5, TBx5, Hand1. Braveheart acts upstream Mesp1 and regulates the temporal activation of cardiac genes through modulation of Mesp1 itself	[27]
		Braveheart interacts with SUZ12 that acts as a histone methyltransferase.	
		Braveheart induces the differentiation of murine bone-marrow-derived mesenchymal cells into cells with a cardiogenic phenotype.It increases sarcomeric α-actin and cardiac troponin T expression and the upregulation of Gata4, Nkx2.5, Isl-1 and Mesp1.	[28]
	Fendrr	Fendrr Interacts with PRC2 and Trg/MLL complex to modulate the chromatin signature of pitX2 and Foxf1.	[29]
		Loss of Fendrr affects the expression of Nkx2.5 and Gata4. Fendrr knockout is embryonic lethal in mice due to defect on the heart septum.	[29]
***MicroRNAs***	miR-1, miR-499	miR-1 controls myogenic differentiation in mouse heart	[30]
		miR-499 is a cardiac specific miRNA	
		miR-1 and miR-499 enhance the cardiac differentiation of cardiomyocyte progenitor cells, probably targeting Sox6 with a consequent increasing of α-cardiac actinin and cardiac troponin T	
		Inhibition of miR-1 and miR-499 blocks cardiac differentiation.	
	miR-322/-503 cluster	miR-322/-503 cluster encodes in an intergenic region on the X-chromosome and increases Nkx2.5, Mef2c, Tbx5, α-MHC inducing CM differentiation, probably targeting Celf1, whereas their deletion reduces the expression of cardiac markers	[33]
		miR-322/-503 cluster acts by the repression of their target Celf1, that lead the ESC to the neuronal differentiation: it is likely that the miR-322/-503 cluster promotes the cardiac differentiation impairing the neuronal through Celf1 inhibition	
	miR-208	miR-208 is involved in the regulation of myosin heavy chain isoform switch during developmental and pathophysiological condition.	[35]
	miR-1-2	miR-1-2 induces cardiac differentiation of murine bone marrow-derived mesenchymal stem cells by Wnt signaling pathway	[34]
		Transfection with miR-1-2 increases expression of Nkx2.5, Gata4, cTnI	
	miR-133	miR-133 together with Gata4, Tbx5 and Mef2c improves cardiac reprogramming from human or murine fibroblast, by repressing Snai1	[30,32]
	miR-26b	miR-26b promotes cardiac differentiation of P19 cells, by regulating canonical and non-canonical Wnt pathway. It represses the expression of Wnt5a and Gsk3β	[31]
	let-7	let-7 family is upregulated during in vitro human cardiac differentiation.	[36]
		The overexpression of members of let-7 family for 2 weeks in hESC derived CMs increases contractile force, cell size, sarcomere length and action potential duration. Knockdown of let-7 results in a reduction of sarcomere length and expression of cardiac maturation markers. Let-7 family probably acts downregulation two of its targets, IRS2 (a member of insulin signaling pathway) and EZH2 (a histone methyltransferase that can regulate gene expression)	

**Table 2:** Overview of selected protocols for in vitro hCMs generation.

Differentiation Condition	Inductive Factors	Beating Starting	Efficiency	CM Subtypes	Functional Assays	Ref
**EB Formation-Based Culture**						
Static suspension culture	Activin A, BMP4, VEGF, DKK1, bFGF, Ascorbic Acid	Day 10	40–50% (cTNT day 14–16)	atrial, ventricular	Extracellular electrical activity, Patch clamp analysis, Cell transplantation	[45]
Static suspension culture	Activin A, BMP4, IWR-1, Ascorbic Acid, Blebbistatin	Day 7	100% beating EBs day 15 90% cTNT day 21	Ventricular	Extracellular electrical activity, Patch clamp analysis, Optical mapping of membrane potential	[46]
Forced aggregation (96 well)	Activin A, BMP4, VEGF, SCF, WNT3a	Day 9	96% beating EBs day 10 27% Nkx2.5 day 10	n.a.	Extracellular electrical activity, Patch clamp analysis	[47]
Forced aggregation (96 well and AggreWell)	Activin A, BMP4, bFGF, Lipids, Insulin, CHIR, IWP-2	Day 6	100% beating EBs day 6 50% cTnT day 6	ventricular	Extracellular electrical activity, Patch clamp analysis, Intracellular calcium transient imaging	[48]
**Monolayer Culture**						
Monolayer	Activin A, BMP4	Day 12	50% MHC day 21	n.a.	Transplantation to the heart	[49]
Monolayer-sandwich	Activin A, BMP4, bFGF	Day 7	90% cTnT day 30	Mixed	Patch clamp analysis, Intracellular calcium transient imaging	[50]
Monolayer	BMP4, bFGF, CHIR, IWP-2, Ascorbic Acid	Day 6	90% cTnT	ventricular	Extracellular electrical activity, Patch clamp analysis, Intracellular calcium transient imaging	[48]
Monolayer	CHIR, IWP-2	Day 7	98% cTnT day 15	Mixed (atrial and ventricular)	Patch clamp analysis	[51]
Monolayer	CHIR, WNT-C59, Ascorbic Acid	Day 7	90% cTnT	Mixed	Extracellular electrical activity-based nanopillar recording, Patch clamp analysis	[52]
Monolayer	CHIR99021, IWR-1, T3, Dexamethasone		80% colocalization of sarcomeric alpha actinin and Junctophilin 2	Mixed	T Tubule staining, Paced Calcium Transients, Calcium Kinetics and contractility	[53]
**Suspension Large Scale Culture**						
Matrix-dependent aggregates/Rocker culture	CHIR, IWP2	Day 7	65% (cTnT/day 12)	n.a.	Toxicology assay	[54]
Matrix-dependent aggregates/EB formation/spinner flasks	SB203580	Day 10	80% (beating EBs/day 16) 20% (MHC/day 16)	n.a.	QT prolongation assay and CM toxicity test	[55]
Matrix-independent aggregated/Erlenmeyer Flask and bioreactor	CHIR, IWP2	Day 6–7	84% (cTnT, MHC/day 10)	80–90% ventricular	Bioartificial cardiac tissue generation, Patch clamp analysis, Extracellular electrical activity	[56]
Matrix-independent aggregated/Spinner flask	CHIR, IWR-1, SB431542, Purmorphamine	Day 7	>90% (cTnT/day 15)	n.a.	Extracellular electrical activity, Patch clamp analysis	[57]

**Table 3:** Physiological characteristics in adult and hiPSC-derived CMs. Adapted from Denning et al. [93].

		Adult-CM	hPSC-CM
Beating		Quiescent	Present
Conduction Properties	Capacitance	150 pF	20–50 pF
	Resting mem potential	−80 to −90 mV	−20 to −60 mV
	Upstroke velocity	150–350 V/s	10–50 V/s
	Conduction velocity	60 cm/s	10–20 cm/s
	Location of gap junctions	Intercalated discs	Circumference of cells
Ion channel density (pA/pF)	I_Na_	−196	−100 to −244
	I_CaL_	−4.3 to −10.2	−2.2 to −10
	I_to_	2.3 to 10.6	2.5 to 13.7
	I_Ks_	0.18 to 0.58	0.3 to 0.7
	I_Kr_	0.5	0.4 to 0.8
	I_K1_	−12	0 to −3.4
	I_NCX_	2.5 to 3	3.6 to 7.9 (inward mode)
Ca^2+^ kinetics	APD90	260 ms	300–700 ms
	Cycle Length	0.8–1 s	0.8–2 s
	T-rise	2.5 ms	3.5–10 ms
	Triangulation	45 ms	45–120 ms

**Table 4:** Cardiac Safety Index of the Tyrosine kinase inhibitors (TIKs). Adapted from Sharma et al. [136].

Drug	Cardiac Safety Index
Afatinib	0.444
Erlotinib	0.635
Gefitinib	0.409
Lapatinib	0.209
Axitinib	1.000
Cabozatinib	0.769
Pazopanib	0.671
Ponatinib	0.483
Regorafenib	0.010
Sorafenib	0.004
Sunitinib	0.218
Vandetanib	0.041
Bosutinib	0.315
Dasatinib	0.524
Imatinib	0.126
Nilotinib	0.104
Dabrafenib	0.459
Vemurafenib	0.003
Trametinib	1.000
Ibrutinib	0.507
Crizotinib	0.063

The cardiac safety index is a value ranging from 0 to 1 that, analyzing the drug effects on both the viability and physiological parameters, provides a relative metric for TKI cardiotoxicity. Drugs with a safety index at or below 0.10 are highly cardiotoxic compounds.
